# A naturally occurring nucleotide polymorphism in the *orf2/folc* promoter is associated with *Streptococcus suis* virulence

**DOI:** 10.1186/s12866-014-0264-9

**Published:** 2014-11-12

**Authors:** Astrid de Greeff, Herma Buys, Jerry M Wells, Hilde E Smith

**Affiliations:** Central Veterinary Institute of Wageningen UR, Edelhertweg 15, 8219, PH Lelystad, The Netherlands; Wageningen UR, Host Microbe Interactions, De Elst 1, 6708, WD Wageningen, The Netherlands

**Keywords:** *Streptococcus suis*, Piglets, Virulence, Pathogenesis

## Abstract

**Background:**

*Streptococcus suis* is a major problem in the swine industry causing meningitis, arthritis and pericarditis in piglets. Pathogenesis of *S. suis* is poorly understood. We previously showed that introduction of a 3 kb genomic fragment from virulent serotype 2 strain 10 into a weakly virulent serotype 2 strain S735, generated a hypervirulent isolate. The 3 kb genomic fragment contained two complete open reading frames (ORF) in an operon-structure of which one ORF showed similarity to folylpolyglutamate synthetase, whereas the function of the second ORF could not be predicted based on database searches for protein similarity.

**Results:**

In this study we demonstrate that introduction of *orf2* from strain 10 into strain S735 is sufficient to dramatically increase the virulence of S735 in pigs. This increase in virulence could not be associated with changes in pro-inflammatory responses of porcine blood mononucleated cells in response to *S. suis in vitro*. Sequence analysis of the *orf2*-*folC*-operon of *S. suis* isolates 10 and S735 revealed an SNP in the −35 region of the putative promoter sequence of the operon, as well as several SNPs resulting in amino acid substitutions in the ORF2 protein. Transcript levels of *orf2* and *folC* were significantly higher in the virulent strain 10 than in the weakly virulent strain S735 and *in vitro* mutagenesis of the *orf2* promoter confirmed that this was due to a SNP in the predicted −35 region upstream of the *orf2* promoter. In this study, we demonstrated that the stronger promoter was present in all virulent and highly virulent *S. suis* isolates included in our study. This highlights a correlation between high *orf2* expression and virulence. Conversely, the weaker promoter was present in isolates known to be weakly pathogenic or non-pathogenic.

**Conclusion:**

In summary, we demonstrate the importance of *orf2* in the virulence of *S. suis.*

**Electronic supplementary material:**

The online version of this article (doi:10.1186/s12866-014-0264-9) contains supplementary material, which is available to authorized users.

## Background

*Streptococcus suis* is a zoonotic pathogen that is ubiquitously present among swine populations in the pig industry. Thirty-three capsular serotypes have been described to date [1] of which serotypes 1, 2, 7, 9 and 14 are frequently isolated from diseased pigs in Europe [2]. Strain virulence differs between serotypes and even within a serotype: virulent, avirulent and weakly virulent isolates have been isolated based on the expression of virulence markers, muramidase released protein (MRP) and extracellular factor (EF) [3] and suilysin [4,5].

Nasopharyngeal carriage of *S. suis* in adult pigs is asymptomatic, whereas in young piglets this increases susceptibility to *S. suis* invasive disease, leading to meningitis, arthritis and serositis, and high rates of mortality [5]. In Western countries humans occupationally exposed to pigs or uncooked pork might also become infected by *S. suis* although the incidence is very low [6]. Invasive *S. suis* infection of humans gives similar clinical signs as in pigs; patients often suffer from remaining deafness after recovery [6]. In Southeast Asia however, *S. suis* is considered an emerging pathogen for humans, and is recognized as leading cause of bacterial meningitis [7-10]. In Southeast Asia, clinical signs of human infections with *S. suis* are reported to be more severe compared to other parts of the world, with patients developing toxic shock-like syndrome, sepsis and meningitis [8].

Previously, a hypervirulent *S. suis* isolate (S735-pCOM1-V[10]) was generated that causes severe toxic shock-like syndrome in piglets after infection resulting in death within 24 h post-infection [11]. S735-pCOM1-V[10] was selected from a library of clones generated in a weakly virulent serotype 2 isolate (S735), after transformation with plasmid DNA isolated from around 30,000 pooled clones carrying randomly cloned genomic DNA fragments from a virulent serotype 2 isolate (strain 10). Isolates with increased virulence were selected by infecting piglets with strain S735 containing the plasmid library of genomic fragments from strain 10. One prevalent clone isolated from the infected piglets contained a 3 kb genomic fragment from strain 10 designated V[10] and was demonstrated to be hypervirulent in subsequent animal experiments. V[10] contained an incomplete open reading frame (ORF), followed by two genes (*orf2* and *folC*) in an operon structure as well as a second incomplete ORF [11]. Assuming that only the full-length ORFs could contribute to the hypervirulence of this isolate, we further characterized the *orf2*-*folC*-operon. The first ORF in the operon could not be annotated and was designated *orf2*, the second ORF in the operon showed homology to polyfolylpolyglutamate synthase (*folC*) [11]. This operon was present in all *S. suis* serotypes, including the parent strain S735. Strain S735 with low virulence, contained several single nucleotide polymorphisms (SNP) in *orf2*-*folC* and the non-coding regions compared to strain 10 [11].

In this study, we aimed to explain the increased virulence of the strain containing the *orf2-folC* operon. We demonstrate that overexpression of *orf2* suffices to increase the virulence, and that a SNP in the predicted −35 region upstream of the promoter of the operon is strongly associated with virulence in *S. suis* isolates. Furthermore, the stronger promoter was shown to be present in all virulent or highly virulent *S. suis* isolates that were included in our study, highlighting a correlation between high *orf2* expression and virulence.

## Results

### Overexpression of *orf2* increases virulence of strain S735

Introduction of a 3 kb genomic fragment from virulent serotype 2 strain 10 increased the virulence of the weakly virulent serotype 2 strain S735 [11], creating a hypervirulent isolate (S735-pCOM1-V[10]). All pigs infected with S735-pCOM1-V[10] died within 1 day post infection (p.i.) and a high percentage of the pigs showed severe clinical signs of disease (Table 1), whereas nearly all pigs infected with the control strain S735-pCOM1 survived throughout the experiment. Clinical indices differed significantly (p ≤0.01) between pigs infected with S735-pCOM1-V[10] and S735-pCOM1 (Table 1). As a control we also tested the virulence of S735 transformed with a plasmid containing the homologous 3 kb fragment from strain S735 (S735-pCOM1-V[S735]). A high percentage of the pigs infected with S735-pCOM1-V[S735] survived throughout the experiment. In contrast pigs infected with S735-pCOM1-V[S735] showed significantly more specific clinical signs (p ≤0.01) than pigs infected with S735-pCOM1 (Table 1), although differences in clinical indices for fever and non-specific symptoms were not significantly different between the groups (p = 0.06). Thus the increased copy number of V[S735] in S735, due to introduction of plasmid pCOM1-V[S735] increased specific clinical signs of *S. suis*. Nevertheless, the specific and non-specific clinical signs due to porcine infection with S735-pCOM1-V[10] (p ≤0.01) were significantly increased compared to pigs infected with S735-pCOM1-V[S735], demonstrating that the introduction of V[10] in strain S735 increased the virulence more than introduction of V[S735]. This result indicated that hypervirulence of strain S735 pCOM-1-V[10] might be due to the different nucleotide polymorphisms in V[10] compared to V[S735].

**Table 1:** **Virulence of complemented** ***S. suis*** **strains in germfree piglets; all strains contained a plasmid (pCOM1) with or without insert**

						**Clinical index of the group**			**No. of pigs in which** ***S. suis*** **was isolated from**		
**Strain**	**No. of pigs**	**Dose (CFU)**	**Mortality** ^**a**^ **(%)**	**Mean no. of days till death**	**Morbidity** ^**b**^ **(%)**	**Specific** ^**c**^ **symptoms**	**Non-specific** ^**d**^ **symptoms**	**Fever index** ^**e**^	**CNS**	**Serosae** ^**g**^	**Joints**
S735-pCOM1-V[10]	4	106	100	1	100	100**	100**	38*	4	4	4
S735-pCOM1-*orf2*[10]	4	106	100	1	100	100**	66**	29	4	4	4
S735-pCOM1-*folC*[10]	4	106	0	11	0	4	21	1	0	0	0
S735-pCOM1	4	106	0	11	0	0	21	5	0	0	0
S735-pCOM1-V[10]f	5	106	100	1	100	100**	100**	60*	5	5	5
S735-pCOM1-V[S735]f	5	106	20	15	100	43**	38	25	1	1	1
S735-pCOM1f	5	106	20	16	60	14	11	12	1	0	0
T15-pCOM1-V[10]	5	106	0	14	16	4	16	13	1	1	1

V[10]/V[S735]: original 3 kb fragment from strain 10 or strain S735 that was selected from library; orf2[10]: orf2 from V[10]; *folC*[10]: orf3 from V[10]encoding dihydrofolate synthase.

^a^Percentage of pigs that died due to infection or had to be killed for animal welfare reasons.

^b^Percentage of pigs with specific symptoms.

^c^Percentage of observations for the experimental group in which specific symptoms (ataxia, lameness of a least one joint and/or stillness) were observed.

^d^Percentage of observations for the experimental group in which non-specific symptoms (inappetite and/or depression) were observed.

^e^Percentage of observations for the experimental group of a body temperature of >40°C.

^**f**^Previous experiments (Smith *et al.*, 2001) were re-analyzed to allow for statistical comparison between experiments, this re-analysis required new stringent definitions of specific and aspecific symptoms as indicated in materials and methods.

*p ≤0.05 compared to S735-pCOM1.

**p ≤0.01 compared to S735-pCOM1.

^g^Serosae are defined as peritoneum, pericardium or pleura.

To determine whether V[10] alone could increase the virulence of an otherwise avirulent *S. suis* strain, pCOM1-V[10] was introduced into the avirulent serotype 2 strain T15 to generate strain T15 pCOM1-V[10]. All piglets infected with T15-pCOM1-V[10] survived throughout the experiment (14 d p.i.), some piglets did show mild clinical signs including fever and specific symptoms (Table 1). Although wild-type isolate T15 was not included into this study, clinical signs or fever have not been previously reported in piglets intranasally infected with this avirulent serotype 2 isolate [12,13]. Based on this results we tentatively concluded that the introduction of pCOM1-V[10] might increase the virulence of strain T15 slightly, although the effect was smaller than that observed in strain S735, and could also be due to intravenous inoculation. Obviously, the genetic background of a strain determines whether introduction of V[10] suffices to increase the virulence tremendously.

To determine if the complete *orf2*-*folC*-operon was required for the observed increase in virulence, both genes of the operon from strain 10 and its cognate promoter were introduced separately into strain S735 to generate strains S735-pCOM1-*orf2*[10] and S735-pCOM1-*folC*[10]. Virulence of these isolates was determined in an experimental infection in piglets, using S735-pCOM1-V[10] and S735-pCOM1 as controls. Table 1 shows that pigs infected with S735-pCOM1-V[10] or with S735-pCOM1-*orf2*[10] died within one day p.i. with severe clinical signs. Infected pigs developed toxic shock-like syndrome that was not observed using wild-type strain 10 in experimental infections, implying fragments V[10] and *orf2*[10] increased virulence of S735 yielding more virulent isolates than strain 10 [3]. Both specific and non-specific symptoms were significantly increased (p <0.01) in pigs infected with S735-pCOM1-V[10] or with S735-pCOM1-*orf2*[10] compared to S735-pCOM1 (Table 1). Bacteriological examination showed that CNS, serosae and joints were colonized by high CFU of *S. suis*. In contrast pigs infected with S735-pCOM1-*folC*[10] or S735-pCOM1 lived throughout the experiment (11 days p.i.) showing mild symptoms of infection, like fever. No significant differences in clinical outcome were observed between pigs infected with S735-pCOM1-*folC*[10] and with S735-pCOM1. This clearly demonstrates that introduction of *folC*[10] does not increase the virulence of strain S735, whereas introduction of V[10] and *orf2*[10] increased the virulence of strain S735. Therefore, we concluded that the observed increased virulence of S735-pCOM1-V[10] compared to S735-pCOM1 was attributed to introduction of *orf2*[10].

### Innate immune response of porcine PBMCs

**Figure 1:**
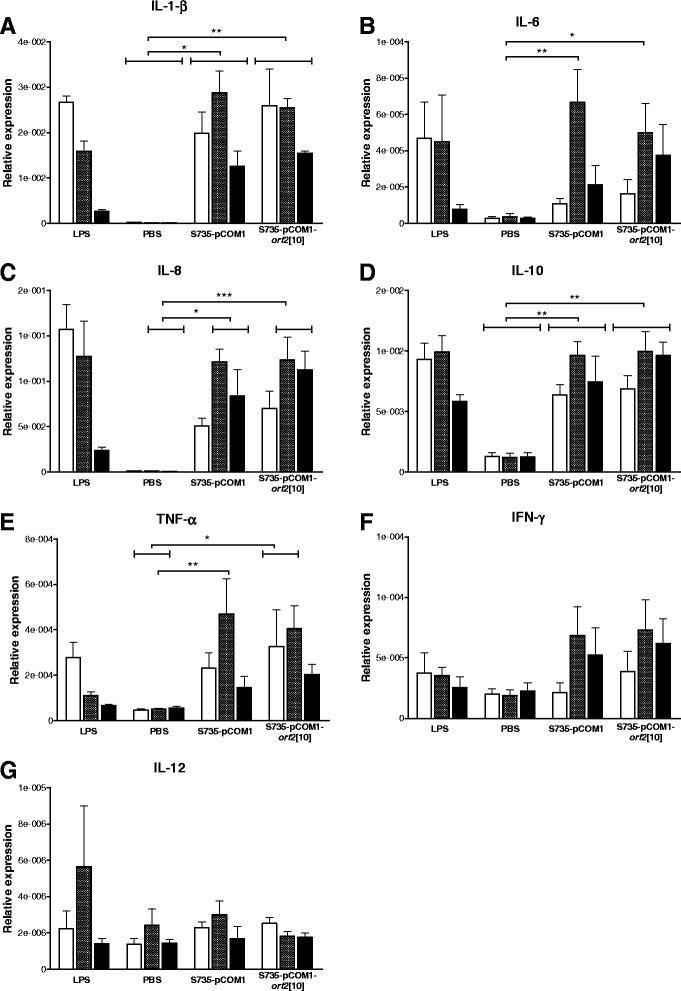
**Innate immune response of porcine PBMCs to** ***S. suis*** **isolates.** Porcine PBMCs were incubated with *S. suis* strains S735-pCOM1 and S735-pCOM1-*orf2*[10] at an MOI of 1. Gene expression of IL-1-β **(panel A)**, IL-6 **(panel B)**, IL-8 **(panel C)**, IL-10 **(panel D)**, TNF-α **(panel E)**, IFN-γ **(panel F)**, and IL-12 **(panel G)** was determined using qPCR after 2 h (white bars), 4 h (hatched bars) and 6 h (black bars) of stimulation. Relative expression was determined by expressing the amount of target gene relative to a housekeeping gene. LPS: lipopolysaccharide; PBS: phosphate buffered saline. Each bar represents two individual experiments each performed in duplo. Error bars represent standard error of the mean. Significance was determined by 2-way ANOVA analysis, only significant differences between PBS treatment, S735-pCOM1 and S735-pCOM1-orf2[10] are indicated, *p <0.05; **p <0.01; ***p <0.001.

As clinical signs of *S. suis* strains S735-pCOM1-V[10] and S735-pCOM1-*orf2*[10] were severe, it was hypothesized that introduction of *orf2* might exacerbate the innate inflammatory response to *S. suis* contributing to the host pathology and symptoms. To test this hypothesis, immune responses of porcine PBMCs to S735-pCOM1-*orf2*[10] and S735-pCOM1 were compared *in vitro*. Gene expression levels of innate immune genes of PBMCs were determined after incubation with *S. suis* isolates as a function of time (2, 4 and 6 h p.i.) using qPCR. Both S735-pCOM1 and S735-pCOM1-*orf2*[10] induced high gene expression of pro-inflammatory cytokine IL-1-β (170-fold) and chemokine IL-8 (130-fold) compared to controls whereas relatively low gene expression of pro-inflammatory IL-6 (15-fold), anti-inflammatory IL-10 (8-fold), and TNF-α (9-fold) and IFN-γ (4-fold) was induced by both isolates (Figure 1). The transcript levels peaked at 4 h p.i. for all genes tested except for IL-12 which was not expressed in response to *S. suis* during the incubation time of our experiment (Figure 1). There were no significant differences between the two isolates in the expression levels of any of the tested immune genes. These data suggest that the observed increased virulence of S735-pCOM1-*orf2*[10] in piglets compared to S735-pCOM1 is probably not due to differences in the host innate responses to this strain.

### A promoter SNP leads to differential expression of the *orf2/folc* operon

**Figure 2:**
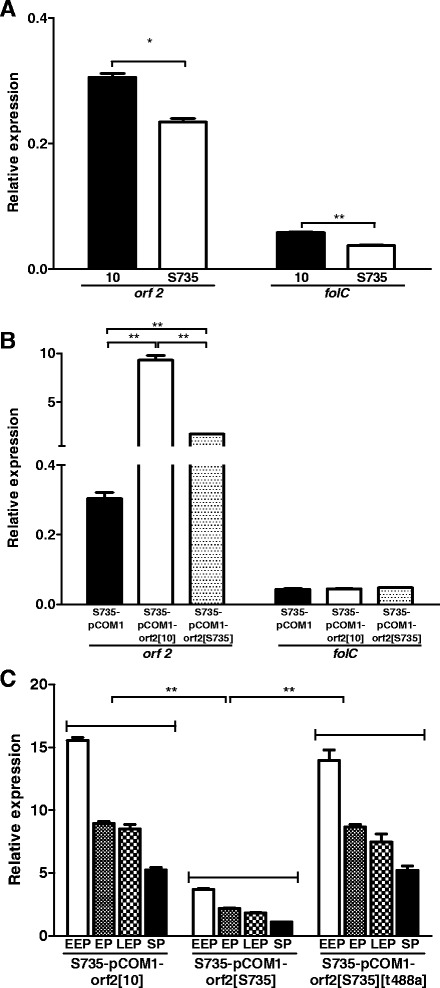
**Expression levels of** ***orf2*** **and** ***folC*** **in** ***S. suis*** **wild-type isolates and mutants.** Expression level of *orf2* and *folC* in *S. suis* wild-type isolates strain 10 (black bars) and S735 (white bars) grown exponentially in Todd Hewitt **(panel A)**; and in strain S735 complemented with empty control plasmid pCOM1 (black bars), with *orf2*[10] (white bars) or with *orf2*[S735] (hatched bars) grown exponentially in Todd Hewitt **(panel B)**. Expression level of *orf2* in S735 complemented with *orf2*[10], *orf2*[S735] and *orf2*[S735][t488a] after growing in Todd Hewitt until early exponential phase (EEP) (white bars), exponential phase (EP) (small hatched bars), late exponential phase (LEP) (large hatched bars) and stationary phase (SP) (black bars) **(panel C)**. Expression levels were determined using qPCR and expressed as relative expression to housekeeping gene *recA*. The experiments were performed in triplicate, error bars indicate standard error of the mean. Significance was determined by paired t-tests. *p <0.05; **p <0.01.

Sequence analysis of the putative promoter of *orf2* revealed a difference at one nucleotide position in the −35 region of the putative promoter in strain 10 (TGG**A**CA) compared to strain S735 (TGG**T**CA) [11]. The effect of this SNP on expression levels of *orf2* and *folC* in strains 10 and S735 was determined using qPCR analysis. Significantly higher levels of expression of *orf2* as well as *folC* were observed in strain 10 compared to strain S735 (Figure 2A). This clearly indicates that the SNP in the −35 region of the putative promoter affects the transcription of *orf2* and *folC*. Thereby, it demonstrates that the identified SNP was indeed located in the promoter region co-transcribing *orf2* and *folC* in an operon. Moreover, introduction of pCOM1-*orf2*[10] into S735 increased expression of *orf2* 31-fold compared to introduction of pCOM1, whereas introduction of pCOM1-*orf2*[S735] increased expression of *orf2* only 5-fold (Figure 2B). As expected expression levels of *folC* were similar for both recombinant strains (Figure 2B). To confirm that the identified SNP in the −35 region of the promoter is responsible for the differences in transcription of *orf2* in strains S735 and 10 the TGG**T**CA of *orf2*[S735] was mutated to TGG**A**CA as found in the promoter of *orf2*[10] (yielding strain S735-pCOM1-*orf2*[S735][t488a]. Transcript levels of *orf2* in S735-pCOM1-*orf2*[S735][t488a] were shown to be similar to that of strain S735-pCOM1-*orf2*[10] and four-fold higher than that of strain S735-pCOM1-*orf2*[S735] in different growth phases (Figure 2C). Both promoters are most active early in the growth phase of *S. suis* when grown in Todd Hewitt broth (Figure 2C). Together, these results clearly demonstrate that in strain 10, the promoter upstream of *orf2*-*folC*-operon is stronger than the promoter upstream of this operon in strain S735, due to an SNP in the −35 region.

### Sequence analysis of the-35 region of the *orf2*/*folC* promoter and *orf2* sequence in different strains and serotypes of *S. suis*

To determine whether the SNP linked to increased expression of *orf2*-*folC* operon was associated with particular clonal types or serotypes of *S. suis* the promoter regions of a large collection of isolates were sequenced (Table 2 & Additional file 1: Table S1). All isolates used were recently characterized and typed by CGH and MLST [14]. Based on the sequence data obtained, isolates could be divided in two main groups (Table 2 & Additional file 1: Table S1). The strong −35 promoter region was exclusively found in serotype 1 and 2 isolates that belonged to CGH cluster A and MLST clonal complex 1 and that expressed the EF-protein. The SNP associated with lower promoter activity was found in serotype 7 and 9 isolates belonging to CGH group B (except for two), which are all negative for the expression of EF, as well as in weakly virulent isolates of serotype 2 belonging to CGH group A/Clonal Complex 1 (CC1) that were positive for the expression of the larger form of EF protein (EF*). There were two exceptions; serotype 7 isolate (C126), that belongs to CC1 but does not express the EF-protein contained the SNP linked to higher promoter activity and serotype 7 isolate (7711) which had a different −35 promoter sequence (TTGTCA) for which the promoter strength is undetermined. In conclusion, only CC1 isolates expressing EF protein (and 1 serotype 7 isolate) contain the SNP linked to strong promoter activity. As isolates of this combination of phenotype and genotype are strongly correlated with virulence [14,15], upstream of *orf2*-*folC*-operon is associated with virulent isolates of *S. suis*.

**Table 2:** **Sequence analysis of the −35 region of the** ***orf2*** **/** ***folC*** **promoter among various** ***S. suis*** **isolates and serotypes** ^**1**^

	**Phenotype**				**−35 promoter sequence (5′- 3′)**		
**Serotype**	**MRP** ^**2**^	**EF** ^**3**^	**CGH cluster** ^**4**^	**Clonal complex**	**TGGACA**	**TGGTCA**	**TTGTCA**
1	-	-	B	13		1/1	
1	S	+	A	1	4/4		
2	-	-	B	16/29/147		6/6	
2	+	-	B	28		1/1	
2	+	*	A	1		7/7	
2	-	*	A	1		1/1	
2	+	+	A	1	9/9		
7	-	-	B	29/1	1/8^5^	6/8	1/8
9	-	-	B	16		2/2	
9	*	-	B	16		6/6	
9	+	-	B	16		1/1	

^1^ *S. suis* isolates were described in de Greeff *et al.* [14].

^2^*indicates an higher molecular weight form of MRP; s indicates a lower molecular weight form of MRP.

^3^*indicates an higher molecular weight form of EF.

^4^All isolates were genotyped using Comparative Genome Hybridization (CGH) [14].

^5^This isolate belongs to clonal complex 1.

“-” indicates absence of the protein; “+” indicates presence of the protein.

**Figure 3:**
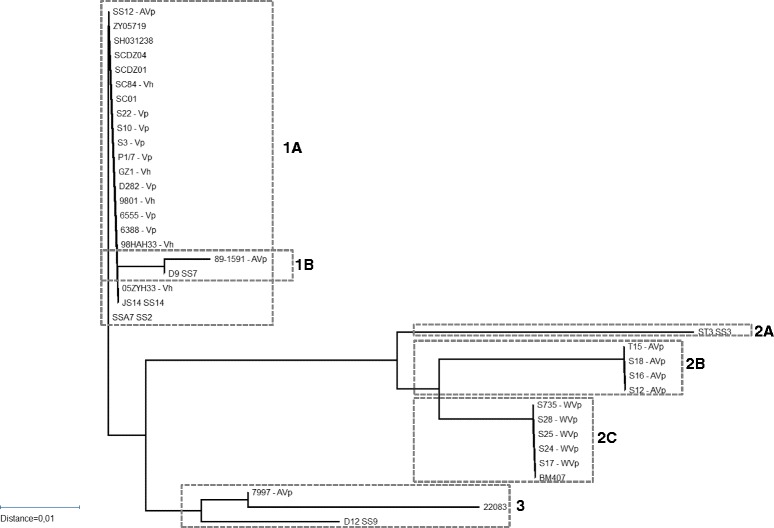
**Alignment of Orf2 sequences of different** ***S. suis*** **isolates as available in the database.** Phylogenetic tree of ORF2 sequences available in the database. Three different clusters are indicated in dotted boxes, sub-clusters are also indicated. Virulence of strains is indicated using V, AV, WV (virulent, weakly virulent, avirulent), as well as the host where virulence was determined indicatd with p or h (pigs, humans).

Besides the correlation between promoter strength and virulence, we also looked for an association between amino acid sequence of ORF2 and virulence. Sequence analysis of the *orf2*-*folC*-operon of wild type strains 10 and S735 revealed several SNPs throughout the sequence [11]. A comparison of ORF2 protein sequences in different isolates revealed more heterogeneity. However, ORF2 protein sequence of all serotype 2 MRP^+^EF^+^ isolates were identical to the sequence of ORF2 in strain 10, besides some variation in the predicted start site of the proteins. Clustering of protein sequences revealed three groups of ORF2 sequences, indicated with cluster 1, 2 and 3 in Figure 3. Within cluster 1, two subclusters could be identified: cluster 1A and 1B. Cluster 1A contained strains with an ORF2 sequence that was identical to strain 10; these isolates seem to be associated with virulence. All of the characterized isolates within cluster 1A belong to CC1 and express EF protein. Furthermore, all isolates containing the stronger promoter belonged to this group. Cluster 1B contained two isolates, a Chinese serotype 7 isolate and a serotype 2 MRP^−^EF^−^ isolate (89–1591), with respectively one and two amino acid substitutions in ORF2 compared to ORF2 of strain 10. The observation that 89–1591 clusters with a serotype 7 isolate, instead of with the other serotype 2 MRP^−^EF^−^ isolates, adds to the speculation that strain 89–1591 is more similar to serotype 7 isolates than to other MRP^−^EF^−^ isolates as was also suggested in our CGH study [14]. Within cluster 2, three subclusters could be identified: cluster 2B contained avirulent serotype 2 isolates that were MRP^−^EF^−^, cluster 2C contained weakly virulent MRP^+^EF^−^ isolates including S735, and cluster 2A contained 1 serotype 3 isolate with unknown virulence. Within these subclusters amino acid sequences are identical, the subclusters differ at 13 (2A), 14 (2B) and 9 (2C) amino acid positions compared to strain 10. Cluster 3 contained serotype 9 isolates from different geographical locations, including the reference strain 5218. In conclusion, isolates that are associated with virulence have a strong promoter and an identical protein sequence of ORF2, whereas less virulent isolates have a weaker promoter and a different protein sequence. The relevance of the heterogenic amino acid composition of ORF2 is unknown, it could interfere with function or folding of ORF2.

## Discussion

In this study we showed that introduction of the *orf2*-gene (*ssu0135* in P1/7) of the *orf2*-*folC*-operon from strain 10 increased the virulence of weakly virulent strain S735, thereby creating a hypervirulent isolate. The increased virulence of S735-pCOM1-V[10] was shown to be correlated with increased expression of *orf2* due to the presence of a stronger promoter in strain 10 compared to strain S735. The promoter of the *orf2*-*folC*-operon differed at single nucleotide position close to the predicted −35 region, implicating that one SNP at a crucial position can increase the virulence of *S. suis* tremendously. The stronger promoter was only present in serotype 1 and 2 isolates belonging to MLST clonal complex 1 that express EF protein, suggesting an association between the strong promoter (and thus higher expression of *orf2*) and virulence.

Introduction of heterologous *orf2*[10] into weakly virulent *S. suis* strain S735 and subsequent infection of pigs with this strain resulted in unusually rapid development of toxic shock-like syndrome and severe clinical signs within 1 day p.i , which resulted in the pigs being euthanized for ethical reasons. Introduction of the homologous fragment containing the intact operon from strain S735 also increased the virulence of the resulting isolate S735-pCOM1-V[S735] significantly compared to the control isolate S735-pCOM1 but the virulence was significantly less that than that of strain S735 pCOM1-V[10]. The virulence of the isolates was correlated with gene expression level of *orf2.* The −35 sequence of the promoter region of the *folC*-*orf2*-operon in strain 10 (TGGACA) deviated from the σ70 consensus promoter (TTGACA) at 1 position, and induced higher expression of both *orf2* and *folC* than the −35 sequence of the promoter region of the *folC*-*orf2*-operon in strain S735 (TGGTCA) that deviates from the σ70 consensus at 2 positions. Introduction of homologous V[S735] preceded by the weaker S735 promoter into strain S735, increased the expression level of *orf2* 5 fold and significantly increased the virulence of the isolate compared to the control S735-pCOM1. Hypervirulence, however, is only achieved when multiple copies of either V[10] or *orf2*[10] both preceded by the stronger promoter were present in strain S735 This demonstrates that increase of heterologous or homologous expression of *orf2* in strain S735, increased the virulence of S735. A similar effect has been observed for the suilysin gene (*sly*), where 2 SNPs in the promoter region of *sly* were detected, and suggested to be associated with more virulent isolates [16]. In *S. suis* a slight increase of virulence occurred after introducing V[10] into avirulent strain T15 although the effect was less pronounced than in strain S735. This suggests that increased heterologous *orf2*[10] expression in strain T15 might increase the virulence, but the completely different genetic background of strain T15 [14,17] probably prohibited a more pronounced increase of virulence of this strain. In conclusion, increased heterologous or homologous expression of *orf2* in either strain S735 or strain T15 was correlated with increased virulence.

Unfortunately, *orf2* did not show homology to any known sequences in the database, so it was not possible to predict the function of *orf2*, and its role in *S. suis* pathogenesis. Based on hydrophobicity plots [11], ORF2 was predicted to be localized in the membrane, and could therefore interact with the host during infection. The host innate response to the hypervirulent isolate was studied *in vitro* in PBMCs*.* A strong pro-inflammatory immune response was induced in PBMCs that peaked 4 h p.i. both after incubation with the hypervirulent isolate as well as with the control isolate (S735-pCOM1). Similar pro-inflammatory host responses to *S. suis* were described for alveolar macrophages [18], brain microvascular cells [19], monocytes [20] and choroid plexus cells [21], suggesting the hypervirulent isolate induced a similar innate response to other *S. suis* isolates *in vitro*. This observation needs to be confirmed *in vivo*.

In post-mortem bacteriology of piglets infected with the hypervirulent isolate, the organs were found to be colonized by extremely large numbers of *S. suis*. This result suggests the hypervirulent isolate can multiply very fast *in vivo*, whereas i*n vitro* the hypervirulent isolate S735-pCOM1-*orf2*[10] showed a slightly decreased growth rate compared to S735-pCOM1 (data not shown). It could have been expected that the high numbers of bacteria present within 24 h in organs of pigs infected with S735-pCOM1-*orf2*[10], resulted in a strong proinflammatory responses, that could ultimately result in the observed toxic shock-like syndrome. However, as *in vitro* measurements did not show any differences in host responses between S735-pCOM1-*orf2*[10] and S735-pCOM1 , it is more likely that introduction of *orf2* into strain S735 is responsible for rapid growth and/or spreading of the bacteria in the host, and is not directly involved in a changed host response. This suggestion could be further substantiated by the *in vitro* expression studies that showed that *orf2* expression is highest in the early exponential growth phase. Although the *in vitro* kinetics of gene expression do not necessarily reflect the *in vivo* kinetics, it is plausible that *orf2* expression is regulated under *in vivo* conditions as well. This would suggest that the advantage of introduction of *orf2* into strain S735 is inducible during the infection process, leading to more efficient breaking of host defence barriers, or to an increased growth rate as was already suggested by the large bacterial load. Recently, a gene homologous to *ssu0135* was annotated as folate transporter *folT* in *S. suis* strain SC070731. This suggests that the whole V[10] operon is involved in folate metabolism. It is known that folate is essential for all living organism including bacteria. Our results would suggest that overexpression of genes involved in folate metabolism would be beneficial for growth and virulence *in vivo*.

In this study, we demonstrated that the stronger promoter was present in all virulent or highly virulent *S. suis* isolates that expressed EF protein that were included in our study, highlighting a correlation between high *orf2* expression and virulence. Conversely, the weaker promoter was present in isolates known to be weakly pathogenic or non-pathogenic. There were some exceptions. A serotype 7 isolate (7711) was shown to have a different promoter that deviated from the σ70 consensus promoter at one position, like the strong promoter. Strength of this promoter is unknown. Another serotype 7 isolate (C126) contained the stronger promoter. This isolate belonged to MLST clonal complex 1, that strictly contains virulent *S. suis* isolates [15] and was isolated from the joints of a lame pig. Based on these observations, it could be speculated that this isolate is more virulent than other serotype 7 isolates. Finally, the serotype 1 reference strain (5482) contained the weak promoter despite being virulent. However, this isolate was already shown not to be a good representative of serotype 1 isolates [14].

## Conclusion

Taken together, the difference in expression level of *orf2* due to different promoter sequences could be responsible for the observed difference in virulence. However, we cannot exclude the possibility that the amino acid substitutions of ORF2 also affect function or effect of the ORF2 proteins of *S. suis* strains. In conclusion, the presence of a strong promoter in the −35 region of the *orf2*-*folC*-operon is associated with virulence. Further research on the biological function of *orf2* may identify novel targets for vaccination or therapy of *S. suis* infections.

## Methods

### Bacterial strains and plasmids

**Figure 4:**
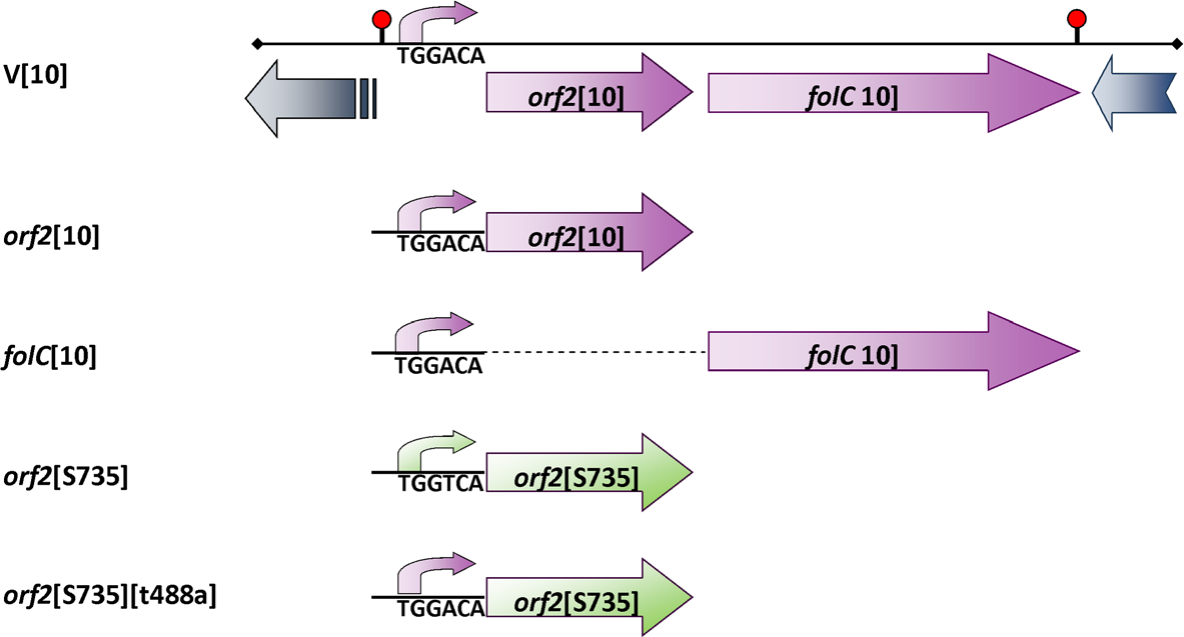
**Constructs used in this study.** V[10] depicts the clone that was identified using a complementation strategy containing two incomplete ORFs (greyish blue arrows) and a putative operon (purple) containing *orf2*[10] and *folC*[10] preceded by the putative promoter of the operon (for clarification only the sequence of the −35 region (TGGACA) of the putative promoter is depicted in the diagram). Constructs were made that contained either *orf2*[10] or *folC*[10] (purple) preceded by the putative −35 region of the putative promoter region of the operon (purple). A construct containing *orf2* from strain S735 (green) with the −35 region of the putative S735 promoter (TGGTCA) (green) was made. The same construct was mutagenized to contain the −35 region of the putative promoter sequence of strain 10 (TGGACA) (purple) yielding *orf2*[735][t488a].

*S. suis* isolates were grown in Todd-Hewitt broth (Oxoid, London, United Kingdom) and plated on Columbia blood base agar plates (Oxoid) containing 6% (vol/vol) horse blood. *Escherichia coli* was grown in Luria Broth and plated on Luria Broth containing 1.5% (wt/vol) agar. If required, erythomycin was added at 1 μg ml^−1^ for *S. suis* and at 200 μg ml^−1^ for *E. coli. S. suis* strain S735 complemented with a plasmid containing a 3 kb genomic fragment derived from strain 10 (S735-pCOM1-V[10]) and the other *S. suis* strains used in this study have been previously described [11,14] (Figure 4).

### Complementation of *S. suis* strain S735

S735 was complemented with plasmid pCOM1 containing one of the two ORFs in the V[10] operon (i.e. *orf2*[10], or *folC*[10]) preceded by the putative promoter region of the operon from strain 10 or with plasmid pCOM1 containing *orf2* and the cognate upstream promoter from strain S735 (*orf2*[S735]) (Figure 4). To construct these plasmids, primers with restriction sites were designed to amplify *orf2*[10] or *orf2*[S735] (comE1 – comE2), *folC*[10] (comE4 – comE6) or the promoter region of the operon (comE1 – comE3) (Table 3). The resulting PCR products *orf2*[10] and *orf2*[S735] were digested using restriction enzymes *Sac*I and *BamH*I, cloned into pKUN19 [22], digested with the same restriction enzymes and subsequently cloned into pCOM1, yielding pCOM1-*orf2*[10] and pCOM1-*orf2*[S735], respectively. The PCR amplicon of *folC*[10] was digested using restriction enzymes *Sm*aI and *BamH*I and cloned into pKUN19 cleaved with the same restriction enzymes. The PCR product comprising the promoter region of V[10] was cloned in front of *folC*[10] using restriction enzymes *Sac*I and *Sma*I. Subsequently, the complete fragment of promoter V[10] – *folC*[10] was digested from pKUN19 using *Sac*I and *Bam*HI and cloned into pCOM1 digested with the same restriction enzymes, yielding pCOM1-*folC*[10]. To confirm that the fusion product of promoter – *folC*[10] was transcribed, *in vitro* transcription/translation was performed using ^35^S-methionine. A clear band of the molecular weight of FolC (46.8 kDa) was detected demonstrating that the fusion product could be expressed and translated. All plasmids were introduced into *S. suis* strain S735 by electroporation. In addition, pCOM1-V[10] was introduced into the avirulent serotype 2 strain T15 [3] by electroporation to yield T15-pCOM1-V[10].

**Table 3:** **Primer sequences**

**Primer name**	**Sequence 5′-3′**	**Target**
comE1	c**gagctc**ggaagaattggttattgcgcgtg	*orf2*[10] – forward - *Sac*I
comE2	cg**ggatcc**cgggggatgacctgttgcttg	*orf2*[10] – reverse – *Bam*HI
comE3	tcc**cccggg**ggagtcgtgtgtattcgacagcgg	P-*orf2-folC*[10] – reverse – *Sma*I
comE4	tcc**cccggg**ggacaagcaacaggtcatcccc	*folC*[10] – forward – *Sma*I
comE6	cg**ggatcc**cggttgaatgcccggcaagcc	*folC*[10] – reverse - *Bam*HI
IL-1-ß-fw	ggccgccaagatataactga	Porcine interleukin 1-ß
IL-1-ß-rev	ggacctctgggtatggctttc	Porcine interleukin 1-ß
IL-6-fw	gacaaagccaccacccctaa	Porcine interleukin 6
IL-6-rev	ctcgttctgtgactgcagcttatc	Porcine interleukin 6
IL-8-fw	ttcgatgccagtgcataaata	Porcine interleukin 8
IL-8-rev	ctgtacaaccttctgcaccca	Porcine interleukin 8
IL-10-fw	gagaaactagggagcccctttg	Porcine interleukin 10
IL-10-rev	tggccacagctttcaagaatg	Porcine interleukin 10
IL-12-fw	ggagtataagaagtacagagtgg	Porcine interleukin 12-p40
IL-12-rev	gatgtccctgatgaagaagc	Porcine interleukin 12-p40
TNF-α-fw	cgcccacgttgtagccaatgt	Porcine TNF-α
TNF-α-rev	cagatagtcgggcaggttgatctc	Porcine TNF-α
IFN-γ-fw	caaagccatcagtgaactcatca	Porcine interferon-γ
IFN-γ-rev	tctctggccttggaacatagtct	Porcine interferon-γ
Orf2-fw	ctacggctggttcttctatcgaa	*S. suis orf2*
Orf2-rev	gcaatcggtgtcatgataaagg	*S. suis orf2*
folC-fw	gtttgtccgtccatcggttt	*S. suis* polyfolylpolyglutamate synthase
Folc-rev	ctggtcggtcgcatagatga	*S. suis* polyfolylpolyglutamate synthase
RecA-fw	ggtttgcaggctcgtatgatg	*S. suis* recombinase A
RecA-rev	accaaacatgacaccgacttttt	*S. suis* recombinase A
t488a	gaaaggtatagtttttagcaagtgg**a**caaaatatatagtgtgtgatacaat	Promoter *orf2*
t488a_antisense	attgtatcacacactatatattttg**t**ccacttgctaaaaactatacctttc	Promoter *orf2*

Bold characters in primers for com indicated the recognition site for restriction enzymes that was added to the primers.

### Experimental infection

Experimental infection of caesarean derived germ-free piglets was performed as previously described [11]. Prior to infection, germ-free status of piglets was confirmed by plating tonsil swabs on Columbia agar plates containing 6% horse blood. Briefly, 4 or 5 one-week-old germ-free pigs were infected intravenously with 10^6^ colony-forming units (CFU) of *S. suis* and then immediately orally administered 40 mg kg^−1^ body weight of erythomycin (erythomycin-stearate, Abbott B.V., Amstelveen, The Netherlands) twice a day to keep selective pressure on *S. suis* isolates harbouring the pCOM plasmids. Infected pigs were monitored twice daily for clinical signs and tonsil swabs collected for bacteriological analysis. Pigs were euthanized when clinical signs of arthritis, meningitis, or sepsis were observed after infection with *S. suis*. Tissue specimens of CNS, serosae and joints were collected during necropsy, homogenized and bacterial cell counts were determined by plating serial dilutions on Columbia agar plates containing 6% horse blood and 1 μg ml^−1^ of erythomycin. To be able to compare results from different animal experiments included in this manuscript, a uniform scoring of non-specific and specific symptoms was applied to all animal experiments. Non-specific symptoms included inappetite and depression that were scored 0 (none), 0.5 (mild inapptite/depression) or 1 (severe inapetite/depression). Specific symptoms included lameness, central nervous system (CNS) symptoms (locomotive disorders like cycling, or walking in circles; opistotonus; nystagmus), as well as raised hairs, arched back (kyphosis), and shivering, since these are all symptoms of sepsis or serositis. Based on these observation clinical indices were calculated by dividing the number of observations where either specific or non-specific symptoms were observed by the total number of observations for this parameter. This represents a percentage of observations where either specific or non-specific symptoms were observed. Fever was defined as a body temperature >40°C. ‘Mean number of days till death’ was used as a survival parameter. Although animals were euthanized after reaching humane end points (HEP), the time between inoculation and reaching HEPs is still indicative of severeness of infection. It is calculated by averaging the survival in days from inoculation until death.

All animal experiments were approved by the ethical committee of the Central Veterinary Institute of Wageningen UR, Lelystad, The Netherlands, in accordance with the Dutch law on animal experiments (#809.47126.04/00/01 & #870.47126.04/01/01).

Statistical analyses were performed on clinical indices of the groups (fever index, specific symptoms and non-specific symptoms) using a non-parametric Kruskal–Wallis test as there was no homogeneity of variance among groups. In subsequent analyses all groups were compared pairwise to the control group (S735-pCOM1) on all three parameters, using Mann–Whitney *U* tests. Differences were considered statistically significant at p <0.05. Calculations were performed using SPSS 19 (IBM, New York, USA).

### Isolation and stimulation of porcine peripheral blood mononuclear cells

Blood from 3 – 4 week old specific pathogen free (SPF) pigs was aseptically collected and mixed with heparin (LEO Pharma, Breda, The Netherlands) to a final concentration of 5 international units (IU) ml^−1^ blood. Blood was subsequently diluted 1:1 with DPBS (Invitrogen, Carlsbad, CA, USA). Peripheral blood mononuclear cells (PBMCs) were isolated using Leucosep tubes (Greiner bio-one, Frickenhausen, Germany) according to manufacturer’s instructions. A cell suspension was generated by passage through a 100 μm cell strainer (BD Falcon, Bedford, MA, USA). PBMCs were washed once in DPBS containing 30 μg ml^−1^ penicillin, and once in RPMI 1640 (Invitrogen) supplemented with 2% of homologous serum (derived from the same donor pig as the PBMCs) and 30 μg ml^−1^ of penicillin. Cells (1 ml) were seeded at a concentration of 5.10^6^ cells per well in 24-well tissue culture plates. After overnight incubation, cells were stimulated with 5.10^6^ CFU exponentially growing *S. suis* cells (multiplicity of infection (MOI) = 1), with 1 μg of lipopolysaccharide as a positive control, or with DPBS as a negative control. Antibiotics were not washed away to increase stimulation of PBMCs as described by Wichgers Scheur [23]. At time point 0 (before stimulation) and after 2, 4, and 6 h of stimulation, the supernatant was removed from the PBMCs and RNA was isolated using Nucleospin RNA II kit (Machery Nagel, Düren, Germany) according to manufacturer’s instructions.

### cDNA synthesis and quantitative PCR

#### RT-PCR

Two hundred ng of RNA was used to synthesize cDNA in a reaction containing 25 ng μl^−1^ random primers (Promega, Madison, WI, USA), 10 mM dNTPs (Promega), 10 mM DTT (Invitrogen), 40 U RNAsin (Promega) and SuperScriptII Reverse Transcriptase (Invitrogen) according to manufacturer’s instructions.

#### qPCR

cDNA was diluted 20 times for qPCR analysis. Primers were designed using PrimerExpress software (Applied Biosystems, Foster City, CA, USA) (Table 3), except for the porcine reference genes that were ordered at GeNorm (Sequenom, San Diego, CA, USA). Each reaction contained 12.5 pmol forward primer, 12.5 pmol reverse primer and POWR SYBR Green PCR Master Mix (Applied Biosystems) according to manufacturer’s instructions. qPCR was performed using an ABI7500 (Applied Biosystems). GeNorm software (GeNorm) was used to determine the most stably expressed reference genes. Of the 5 candidate reference genes tested for porcine RNA, the transcript amounts of *ppiA* and *gp1* were the least variable between the different samples. For *S. suis recA* was the least variable in expression of the 6 potential reference genes (phosphogelycerate dehydrogenase (*pgd*)*,* acetyl coA acetyltransferase (*aca*)*, mutS,* glutamate dehydrogenase (*gdh*), gyrase B) tested. Genorm combines expression data into a number, representing stability of expression, where 1 represents the most stabile gene. Stability numbers for *S. suis* ranged from 1.667 for *gdh* to 1.217 for *recA*. The level of expression of these reference genes was measured to control for variation in RNA-yield and RT-reaction conditions. In each qPCR run a standard curve was incorporated consisting of a vector containing a cloned PCR product of the target gene of that reaction. The standard curve consisted of seven 10-fold dilutions of the control vector. In this way both the expression level of the target gene and the expression levels of external reference genes could be calculated from a standard curve. For each reaction water was included in place of cDNA or template as a negative control. Analysis was performed using the ABI7500 Software (Applied Biosystems).

### Sequence analysis

Sequence reactions were performed by Baseclear (Leiden, The Netherlands).

### Site-directed mutagenesis

Site directed mutagenesis was achieved using the Quick-change II site-directed mutagenesis kit (Agilent Technologies, La Jolla, CA, USA) according to manufacturer’s instructions. PCR primers were designed with the accompanying software (Agilent Technologies) (Table 3). Using primers t448a and t488a_antisense the plasmid pCOM-*orf2*[S735] was amplified, introducing the desired mutation that changed the −35 region of the putative promotor region of the *orf2*-*folC*-operon of S735 from 5′-TGGTCA-3′ to 5′-TGGACA-3′ (Figure 4). The reaction mixture was digested using *Dpn*I to inactivate the original template vector and subsequently transformed to XL-1-blue competent cells (Invitrogen). To exclude the possibility of introducing PCR errors into the vector backbone, the insert of the plasmid (*orf2*[S735]) was isolated from the template vector after digestion with restriction enzymes *Bam*HI and *Sac*I and cloned into pCOM1 digested with the same restriction enzymes. The resulting plasmid was introduced into *S. suis* isolate S735 by electroporation and transformants were selected on Columbia agar containing 1 μg ml^−1^ erythomycin, yielding S735-pCOM1-*orf2*[S735][t488a]. Sequencing was used to exclude presence of PCR errors in the final construct.

## Additional file

Additional file 1: Table S1. Promoter sequence for *Streptococcus suis* isolates of different serotypes and phenotypes.
