# Supplementary material for: A naturally occurring nucleotide polymorphism in the orf2/folc promoter is associated with Streptococcus suis virulence
Source: BMC Microbiol. 2014 Nov 12;14:264. doi: 10.1186/s12866-014-0264-9 (PMC4232619; doi:10.1186/s12866-014-0264-9)
Supplement: Additional file 1: Table S1. — Promoter sequence for Streptococcus suis isolates of different serotypes and phenotypes. [file 12866_2014_264_MOESM1_ESM.docx]

**Supplementary Table:** Promoter sequence for *Streptococcus suis* isolates of different serotypes and phenotypes.

| Isolate | Ss^1^ | Protein Expression of: | | | MLST TYPE^5^ | | PROMOTER SEQUENCE |
| --- | --- | --- | --- | --- | --- | --- | --- |
|  |  | **MRP^2^** | **EF^3^** | **SLY^4^** | **ST** | **CC** |  |
| 6388 | 1 | s | + | + | 1 | 1 | TGG**A**CA |
| 6112 | 1 | s | + | + | 1 | 1 | TGG**A**CA |
| C160 | 1 | s | + | + | 1 | 1 | TGG**A**CA |
| NCTC 10237 / 5428 | 1 | - | - | + | 13 | 13 | TGG**T**CA |
| C187 | 1 | s | + | + | 132 | 1 | TGG**A**CA |
| 3995 | 2 | + | * | - | 1 | 1 | TGG**T**CA |
| 3988 | 2 | + | * | - | 1 | 1 | TGG**T**CA |
| S735 | 2 | + | * | + | 1 | 1 | TGG**T**CA |
| 3 | 2 | + | + | + | 1 | 1 | TGG**A**CA |
| 10 | 2 | + | + | + | 1 | 1 | TGG**A**CA |
| 22 | 2 | + | + | + | 1 | 1 | TGG**A**CA |
| D282 | 2 | + | + | + | 1 | 1 | TGG**A**CA |
| 7696 | 2 | + | + | + | 1 | 1 | TGG**A**CA |
| P1/7 | 2 | + | + | + | 1 | 1 | TGG**A**CA |
| BM191 | 2 | + | * | + | 1 | 1 | TGGTCA |
| BM334 (B) | 2 | - | + | + | 1 | 1 | TGG**A**CA |
| BM407 | 2 | - | * | + | 1 | 1 | TGG**T**CA |
| FX59 (4) | 2 | - | + | + | 1 | 1 | TGG**A**CA |
| 95-8242 | 2 | + | + | + | 1 | 1 | ND |
| R75 / S2 | 2 | + | + | + | 1 | 1 | TGG**A**CA |
| 98HAH12 | 2 | + | + | + | 7 | 1 | TGG**A**CA |
| 05ZYH33 | 2 | + | + | + | 7 | 1 | TGG**A**CA |
| 17 | 2 | + | * | + | 8 | 1 | TGG**T**CA |
| 12 | 2 | - | - | + | 19 | 87 | TGG**T**CA |
| 16/T129 | 2 | - | - | +/- | 19 | 87 | TGG**T**CA |
| T15 | 2 | - | - | + | 19 | 87 | TGG**T**CA |
| 25 | 2 | - | - | + | 20 | 17 | TGG**T**CA |
| 89/1591 | 2 | - | - | - | 25 | 29 | TGG**T**CA |
| 89-999 | 2 | - | - | - | 25 | 29 | TGG**T**CA |
| FX125 (2) | 2 | + | - | - | 28 | 28 | TGG**T**CA |
| 1890 | 2 | + | * | + | 134 | 1 | TGG**T**CA |
| BM190 | 2 | + | * | + | 1 | 1 | TGG**T**CA |
| C126 | 7 | - | - | - | 1 | 1 | TGG**A**CA |
| 7711 | 7 | - | - | - | 29 | 29 | T**T**G**T**CA |
| 7917 | 7 | - | - | - | 29 | 29 | TGG**T**CA |
| 87 | 7 | - | - | - | 29 | 29 | TGG**T**CA |
| 106 | 7 | - | - | - | 29 | 29 | TGG**T**CA |
| 8074 | 7 | - | - | + | 29 | 29 | TGG**T**CA |
| 15009 | 7 | - | - | - | 89 | 87 | TGG**T**CA |
| 8039 | 7 | - | - | - | 135 | 29 | TGG**T**CA |
| 7997 | 9 | * | - | + | 16 | 87 | TGG**T**CA |
| 7709 | 9 | * | - | + | 16 | 87 | TGG**T**CA |
| C132 | 9 | * | - | + | 16 | 87 | TGG**T**CA |
| 7998 | 9 | + | - | + | 16 | 87 | TGG**T**CA |
| 22083 | 9 | * | - | - | 82 | 82 | TGG**T**CA |
| 8067 | 9 | - | - | + | 136 | 87 | TGG**T**CA |
| 8017 | 9 | - | - | + | 136 | 88 | TGG**T**CA |
| 5973 | 9 | * | - | - | 137 | 87 | TGG**T**CA |
| 8186 | 9 | * | - | - | 138 | 138 | TGG**T**CA |
| 2840 | PA | - | * | + | 133 | 133 | TGG**T**CA |

^1^ Serotype as determined by slide agglutination test

^2^ MRP = Muramidase Released Protein; protein expression was detected using monoclonal antibodies on a Western blot; s small variant, * large variant, + positive, - negative

^3^ EF = Extracellular Factor; protein expression was detected using monoclonal antibodies on a Western blot; * large variant, + positive, - negative

^3^ SLY = Suilysin; protein expression was detected using monoclonal antibodies on a Western blot; +/- weakly positive, + positive, - negative

^4^ MLST = Multi Locus Sequence Typing; ST = sequence type; CC clonal complex type
